# Smoking among Hong Kong Chinese women: behavior, attitudes and experience

**DOI:** 10.1186/s12889-015-1529-4

**Published:** 2015-02-25

**Authors:** Ho Cheung William Li, Sophia SC Chan, Tai Hing Lam

**Affiliations:** School of Nursing, The University of Hong Kong, 4/F, William M. W. Mong Block, 21 Sassoon Road, Pokfulam, Hong Kong, China; School of Public Health, The University of Hong Kong, 5/F, William M. W. Mong Block, 21 Sassoon Road, Pokfulam, Hong Kong, China

**Keywords:** Attitude, Behaviour, Chinese, Public health care, Smoking, Women smokers

## Abstract

**Background:**

The numbers of women smoking have risen 72.5% since 1990 with the increasing population – from 56,100 to 96,800 in 2012, reflecting an alarming situation in Hong Kong. The study aimed to describe the smoking behaviour, attitudes and associated factors among women in Hong Kong.

**Methods:**

A qualitative cross-sectional study involving semi-structured interview was conducted with Chinese women from five community centres in different districts in Hong Kong in 2010. A purposive sample of 73 female participants (24 current smokers, 20 ex-smokers and 29 never-smokers) were recruited. The 73 women were classified by their smoking status and age to form 15 focus groups.

**Results:**

Most informants knew about the general health hazards of smoking, such as cancer and heart or respiratory diseases, but not about the female-specific health consequences of smoking. A few smokers considered smoking to be a weight control strategy, fearing a gain in weight if they gave up. Moreover, a few relied on smoking as a coping strategy to relieve negative emotions and stress. Additionally, a few smokers had misconceptions about giving up: that a loss of concentration would result, that continued smoking would not further affect their health as they had become desensitised to the chemicals in tobacco smoke or that quitting would harm their health.

**Conclusions:**

This study generates new knowledge about the behavior, attitudes, and experiences related to smoking of current female smokers, ex-smokers and non-smokers in Hong Kong, which is unique as a Chinese but highly westernized community but with a very low female smoking prevalence.

## Background

Cigarette smoking is the most important preventable cause of death and disease, causing six million deaths annually worldwide [1]. Increasing evidence shows that smoking has negative effects on nearly every organ of the body [2].

The current global population of smoking women is far less than that of men [1]. However, while the epidemic of tobacco use in men is in slow decline, there is growing concern about increasing tobacco use in women [3,4]. It is predicted that 20% of women worldwide will be smokers by 2025, compared with 12% in 2010 [5]. Smoking causes many fatal diseases and presents a large health threat to women [6,7]. Some health consequences of smoking are specific to women, such as a higher rate of infertility, premature labour, low birth weight infants, ectopic pregnancy, sudden infant death syndrome, cervical cancer, irregular menstruation cycles, dysmenorrhoea and early menopause [2,6]. Studies have shown that half those smokers who continue to smoke will be killed by tobacco prematurely [8,9], and the number of deaths among women attributable to tobacco will increase.

Women’s smoking prevalence rates have been rising in Western Europe, Australia and the United States since the beginning of the 20^th^ century [10], and today’s women in these countries smoke at nearly the same rate as men [1]. This increased smoking prevalence among women might well be attributed not just to the changes in social roles and socio-economic status of women, but also to the massive advertising and promotional activities of the tobacco industry to promote smoking as a symbol of emancipation in the 20^th^ century [11,12]. Most women in these countries view smoking as a sign of sophistication and independence [12]. Tobacco advertising is one of the strongest risk factors in smoking initiation among children and adolescents [13,14].

**Figure 1:**
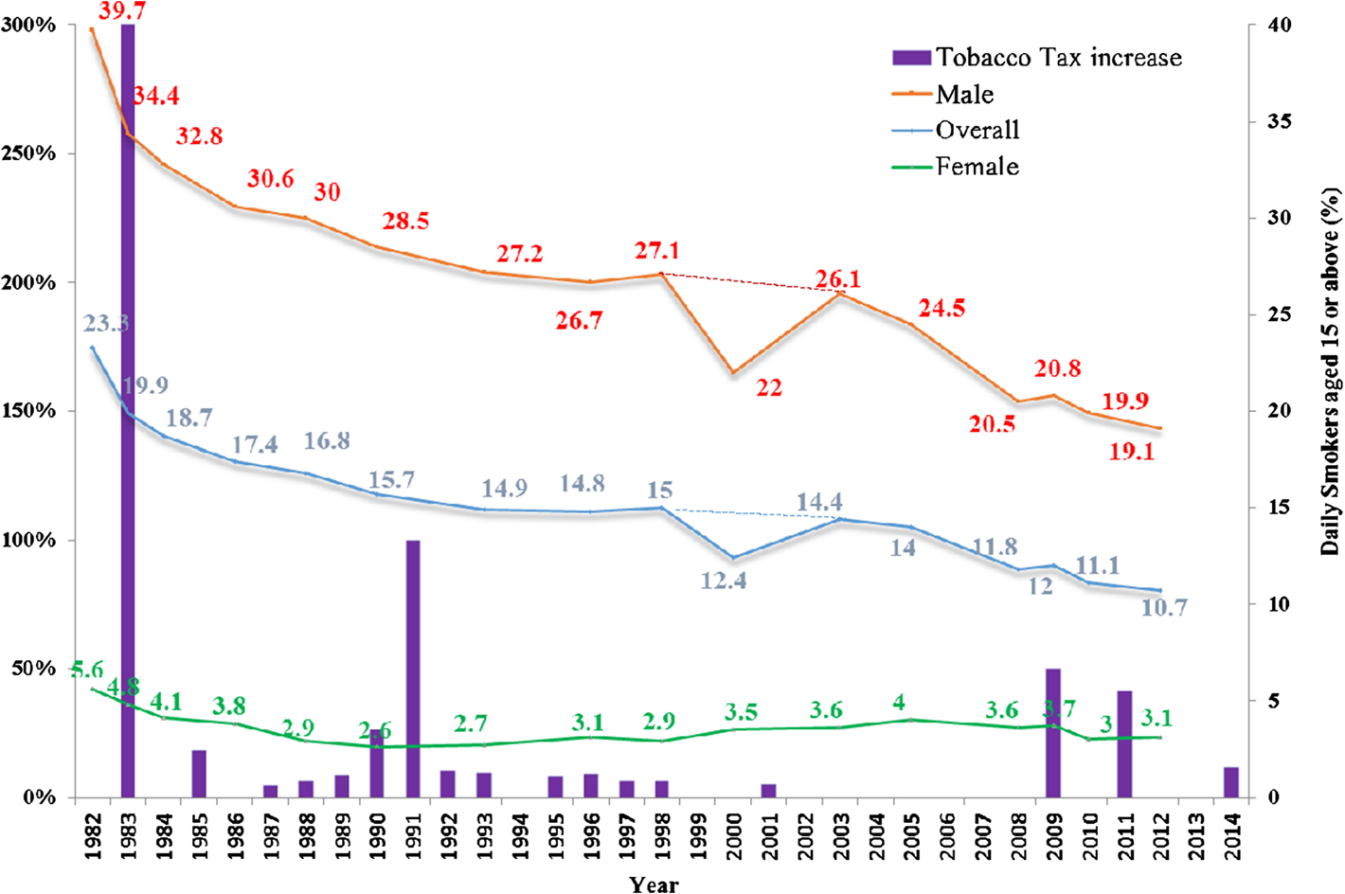
**Prevalence of daily cigarette smokers by sex and a progressive approach to raising tobacco tax in Hong Kong from 1982 to 2014.** Remarks: 1982-1998: General Household Survey conducted by government Census & Statistics Department 2000 onwards: Thematic Household Survey (THS) conducted by contractors (commercial survey companies). The 2000 data, from the first THS appeared to be incredible, as there were no reasons to explain the great declines in men and total. The dotted lines should be more reliable.

Hong Kong, as a former British colony, has been widely influenced by Western culture, and is the most Westernised city in China [15,16]. Given the wide exposure to Western culture, a high smoking uptake rate in Hong Kong Chinese women is to be expected. However, the prevalence of smoking among women in Hong Kong is consistently lower and has never been higher than in most Western countries [1,17]. This very low prevalence in women has contributed to the lowest overall prevalence of daily cigarette smokers aged 15 years or older in the developed world [18]. The reasons for the low prevalence of cigarette smoking in Hong Kong Chinese women might be generally attributed to the enormous efforts of the Hong Kong government and tobacco control advocates in raising tobacco tax, introducing legislation, law enforcement and anti-smoking campaigns over the past 30 years, which have led to remarkable success in tobacco control [19,20]. Since 1982, Hong Kong has taken a progressive approach to tobacco control, raising tobacco tax (Figure 1) and imposing a comprehensive ban in 1999 on tobacco advertising and promotional activities [20]. Furthermore, a smoking ban has been implemented at all indoor restaurants and workplaces and many public places since 2007, extended to clubs and bars in 2009 and further extended to over 129 open-air and two covered public transport facilities in 2010 [21]. Hong Kong has fully aligned itself with the MPOWER measures advocated by the World Health Organisation, and is the only city in China to have established a strong cessation programme ahead of national policy [1,19].

Despite the general low prevalence of smoking, the 96,800 women who are daily smokers in Hong Kong cannot be overlooked or unconsidered [18]. Of them, about half have not attempted or do not want to quit smoking, and 40.0% have attempted but failed [18]. However, while women are still much less likely to smoke than men, their numbers have risen 72.5% since 1990 (Figure 1) with the increasing population - from 56,100 to 96,800, reflecting an alarming situation related to smoking women in Hong Kong. Most importantly, the tobacco industry is actively seeking new customers to replace those who have already quit smoking or who will die prematurely [22]. In China particularly, tobacco marketing campaigns have begun to target women aggressively by promoting smoking as a symbol of emancipation, independence and charisma [23,24], and more effective smoking cessation interventions for female smokers are urgently needed.

Our review of the literature reveals that there is a lack of population-based smoking cessation interventions targeting Chinese woman smokers. Despite increasing concerns, information on smoking and its associated factors in adult women, particularly in Chinese populations, is scarce [24]. No study has evaluated the effectiveness of smoking cessation interventions designed specifically for Chinese women. Data on public awareness of the diseases caused by smoking among Chinese women in Hong Kong, particularly in China are scare. Moreover, a previous study [25] found that the psychological and social factors causing Chinese women to start and continue smoking are complex, and that they might encounter more difficulties and have less confidence in quitting than men. It is of paramount importance to design tailor-made interventions to communicate clearly to women the risks of continued smoking and to motivate them to quit [26]. To support such interventions, it is essential to gain a better understanding of women’s reasons for starting to smoke and for trying or not trying to give up. In particular, the behaviour, attitudes and experiences related to smoking and smoking cessation should be examined. This study aimed to describe the smoking behaviour, attitudes and associated factors among women in Hong Kong.

## Methods

### Design

A qualitative research design was used to study a purposive sample of 73 female participants (current smokers: n = 24; ex-smokers: n = 20; never-smokers: n = 29) recruited from July to September 2010.

### Participants

Purposive sampling was used to maximise the sample variation, allowing us to obtain representative informants for the interviews [27]. The participants were recruited from five community centres in different districts in Hong Kong (Wan Chai, Chai Wan, Mong Kwok, Tuen Mun and Kwai Chung) to increase the generalizability of the findings. To identify potential subjects, advertisements containing details of the study were posted on the notice board at the community centres. If women were interested in the research, they could contact the research assistant via telephone to convey their willingness to participate in the study. They were eligible if they were Hong Kong Chinese residents, aged 15 to 60 years old, had smoked weekly in the past six months or had quit smoking or had never smoked, and could communicate in Cantonese. We excluded those who were not able to communicate effectively due to psychological or mental health reasons.

The 73 women were classified by their smoking status (current smokers, ex-smokers and non-smokers) and age (15–19, 20–29, 30–39, 40–49 and ≥ 50 years old) to form 15 focus groups. Each focus group consisted of four to six participants with similar smoking status and within the same age range.

### Data collection

Approval for the study was obtained from the Institutional Review Board of the University of Hong Kong/Hospital Authority Hong Kong West Cluster. The eligible subjects were invited to participate in the study after they were told its purpose. They were given the option of participating or refusing and were told that their participation was voluntary without prejudice. Written consent was obtained from all participants. For those who were under 18, written informed consent was obtained from their parents or guardians.

Before the interviews, the participants were invited to complete a one-page questionnaire on their demographic and socio-economic characteristics and were asked about their smoking history. Based on their smoking status, we used tailored semi-structured interview guides to guide each focus group. Questions for the non-smoking participants targeted their perceptions about the non-smoking status of females, health-related issues, factors for not smoking and the perceived factors for other females who do not smoke. The current and ex-smokers were asked about their perceptions of smoking among females, knowledge of health-related issues, factors that affected their uptake of smoking and their quitting experiences. An audiotaped semi-structured in-depth interview was conducted with each group. Each group interview lasted about 90 minutes and data saturation was achieved after 15 group interviews.

The interviews were conducted at a quiet venue at the university by the same two research assistants, who had considerable experience in conducting qualitative interviews. The interviews began with a general question and followed by specific questions to encourage the informants to provide more descriptions of each episode. During each interview, one research assistant acted as the interviewer to encourage the informants to freely express their feelings, thoughts and ideas. The second research assistant acted as an observer and documented any non-verbal language used by the informants.

The semi-structured interview guide was developed by the research team, which included two professors with extensive experience and knowledge in conducting research related to smoking, a postdoctoral fellow with experience in conducting qualitative studies and two research assistants. The interview guide was assessed for its relevancy and the appropriateness of its wording by two nurse counsellors with considerable experience in providing smoking cessation counselling. The interview guide was found to be relevant and appropriate and no amendment was required.

### Data analysis

After the interviews were completed, the recordings were fully transcribed, verbatim, in Cantonese to capture nuances of expression unique to the dialect, and selected quotations relevant to the themes were later translated into English. In the coding process, two researchers were responsible for analyzing the narratives. The analyses began with an intensive examination of the transcriptions to search for general constructs and themes. Special attention was given to constructs that diverged from the major topics as framed by the guiding questions.

The transcriptions were first coded using the open coding method. Details in the interview conversations were closely examined to allow a large number of initial categories to emerge. As the number of codes grew, some closely related codes were merged, resulting in a smaller, more manageable set of codes. Selective coding was then adopted to code the transcriptions using the established categories. To facilitate the data analysis process, meetings were held to discuss emergent themes. During the coding process, any inconsistencies in the interpretation of quotations or the assignment of codes were resolved through discussions with the research team members. Finally, a complete set of codes was generated to facilitate comparisons and the development of themes and categories.

To achieve a more coherent and logical structure, the themes and categories were modified by breaking down concepts that were complicated, merging similar ones, and rearranging certain themes and categories.

To ensure data credibility, the interviewer asked iterative questions and used probes during the interviews. Debriefing sessions were held between the research assistants and the principal investigator after interviewing every three groups. Modifications were made by the principal investigator according to the developing ideas and interpretations. The data analysis was performed by two researchers independently and field notes were taken into account in the analysis. Regular research team meetings were held to resolve any disagreements.

To ensure the confirmability and dependability of the findings, an audit trail was conducted by another experienced researcher who did not belong to this research team. She reviewed a collection of documents that attested to the researchers’ interpretations. No queries or disagreements were raised during this process.

## Results

### Demographic characteristics

Table 1 shows that 65.8% of the 73 participants were single, divorced or widowed, 50.7% had children, 90.4% had high school education or above and 28.8% were employed.

**Table 1:** **Socio-demographic characteristics of non-smokers, ex-smokers and smokers (n = 73)**

	**Smoking Status**			
	**Current smoker (n = 24)**	**Ex-smoker (n = 20)**	**Never smoker (n = 29)**	**Total (n = 73)**
	**n (%)**	**n (%)**	**n (%)**	**n (%)**
Age (years**)**				
15 to 19	7 (29.2)	4 (20.0)	6 (20.7)	17 (23.3)
20 to 29	3 (12.5)	5 (25.0)	5 (17.2)	13 (17.8)
30 to 39	4 (16.7)	5 (25.0)	6 (20.7)	15 (20.5)
40 to 49	4 (16.7)	2 (10.0)	6 (20.7)	12 (16.4)
≥ 50	6 (25.0)	4 (20.0)	6 (20.7)	16 (21.9)
Marital status				
Married/partnered	9 (37.5)	6 (30.0)	10 (34.5)	25 (34.2)
Single/divorced/widowed	15 (62.5)	14 (70.0)	19 (65.5)	48 (65.8)
Children				
Yes	15 (62.5)	10 (50.0)	12 (41.4)	37 (50.7)
No	9 (37.5)	10 (50.0)	17 (58.6)	36 (49.3)
Education attainment				
Junior school and below	3 (12.5)	1 (5.0)	3 (10.3)	7 (9.6)
High school	21 (87.5)	14 (70.0)	11 (37.9)	46 (63.0)
College and above	0 (0)	5 (25.0)	15 (51.8)	20 (27.4)
Employment status				
Full time/part time	4 (16.7)	0 (0)	17 (58.6)	21 (28.8)
Unemployed	2 (8.3)	4 (20.0)	0 (0)	6 (8.2)
Full time students	6 (25.0)	6 (30.0)	6 (20.7)	18 (24.7)
Retired/others (e.g., housewife)	12 (50.0)	10 (50.0)	6 (20.7)	28 (38.4)
Residential area				
Hong Kong Island	11 (45.8)	9 (45.0)	4 (13.8)	24 (32.9)
Kowloon	0 (0)	0 (0)	9 (31.0)	9 (12.3)
New Territories	13 (54.2)	11(55.0)	16 (55.2)	40 (54.8)

### Themes

Eight themes were generated by the 15 focus group interviews, with each theme divided into categories: T1, smoking behaviour; T2, factors influencing smoking initiation; T3, factors influencing continued tobacco use; T4, reasons for not starting smoking in never-smokers; T5, reasons for quitting among ex-smokers; T6, knowledge of the adverse effects of smoking on health; T7, perspectives on woman smoking; T8, perspectives on smoking cessation promotion and anti-smoking legislation.

### T1. Smoking behaviour

The majority of current and ex-smokers reported that they had started smoking at a young age, with only a few beginning as adults.

### T2. Factors affecting smoking initiation

Most current and ex-smokers claimed that they started smoking because they had friends, particular their best male or female friends, who smoked and encouraged them to do so too. In addition, parent or sibling smokers also appeared to be a strong determinant of the onset of smoking among ever-smokers. Moreover, relief of negative moods, rebelliousness, curiosity in adolescence and unawareness of the addictive nature of smoking were common factors affecting initiation. Additionally, a few informants reported that smoking made them look ‘cool’ and seem more mature or adult.

### T3. Factors influencing continued tobacco use

The majority of current smokers continued smoking because of peer influence, socialisation and enhanced friendship. They also claimed that smoking had become a habit. Apart from that, a few smokers emphasised the societal pressure to be slim and considered smoking to be a weight control strategy, fearing a gain in weight if they gave up. A few relied on smoking as a coping strategy to relieve negative emotions and stress. Additionally, a few smokers had misconceptions about giving up: that a loss of concentration would result, that continued smoking would not further affect their health as they had become desensitised to the chemicals in tobacco smoke or that quitting would harm their health.

### T4. Reasons for not starting smoking in never-smokers

Most never-smokers perceived strong opposition to smoking from their families, and the adverse effects of smoking on the next generation were a common concern for never-smokers, preventing them from ever starting to smoke. Furthermore, the issues most frequently talked about by never-smokers in the interviews were their concerns about the health hazards of smoking, negative perceptions of the smell of cigarettes and the poor social image of woman smokers.

### T5. Reasons for quitting among ex-smokers

The most common reason for quitting was awareness of the health dangers to others, in particular their babies during pregnancy and breast-feeding. Another very common reason raised by the informants was health concerns, especially when they were diagnosed with an illness or if a relative or friend had developed cancer or died from cancer as a result of smoking. A few ex-smokers endorsed a change in appearance as the key factor in their decision to quit. A further few perceived that woman smoking was generally unacceptable in Chinese society and that they quit because of their boyfriends or because they were looking for a potential dating partner. In addition, a few ex-smokers from lower income groups said that they quit because of the increasing tax on cigarettes and that they could not afford to buy a pack a day.

### T6. Knowledge of the adverse effects of smoking on health

Most current smokers, ex-smokers and non-smokers were aware of the health consequences causally linked to smoking, such as heart and respiratory diseases and lung cancer. However, most were not aware of the female-specific health problems induced by smoking, such as higher rates of infertility, ectopic pregnancy, premature labour, dysmenorrhoea, infants’ low birth-weight, early menopause, osteoporosis and even cervical cancer.

### T7. Perspectives on woman smoking

We observed two very different views of the values and perceived social norms of smoking among never-smokers and current smokers. The never-smokers mostly grew up in non-smoking families and had non-smoking friends. With the influence of their families and friends, they considered woman smoking as socially unacceptable and a violation of Chinese culture and tradition. They perceived women smoking as carrying a stigma, that women smoking was something bad and evil.

Most current smokers grew up with their fathers or male friends smoking and so had a high chance of closely observing people smoking. They were more likely to perceive smoking as a social norm and as a tool for communication and connecting with male friends.

### T8. Perspectives on smoking cessation promotion and anti-smoking legislation

Most current, ex- and never-smokers thought that there were not enough smoking cessation advertisements targeting female smokers. Most current and ex-smokers were also aware of the pictorial warnings on cigarette packets and they felt that the pictures elicited varying degrees of horror and disgust. The majority of never-smokers complained that the publicity on smoking cessation was not as strong as that on the prevention of drug abuse.

The majority of ex-smokers and never-smokers supported raising tobacco tax, and believed that it could reduce smokers’ consumption. A few current smokers said that if the government increased the tax or if the tobacco price was high, then they would consider consuming less or even quitting. Most never-smokers perceived that the smoking ban in places like restaurants and other indoor area was effective. Nevertheless, they queried how well the smoke-free legislation was implemented and suggested that law enforcement was insufficient.

## Discussion

To the best of our knowledge, this is the first report that has examined the behavior, attitudes and experiences related to smoking among Chinese current female smokers, ex-smokers and non-smokers. Given the wide exposure to Western culture but very low prevalence rates of female smoking in Hong Kong, research into the factors affecting cigarette smoking or not smoking in this special population is therefore essential.

### Implications for prevention and clinical practice

The findings revealed that parents who smoke might have a strong negative influence on their children. Healthcare professionals must advise parents that if they do not want their children to smoke they must set a good example by abstaining from smoking. Education programmes are needed to strengthen people’s ability to resist peer influence and curiosity, and to prevent smoking initiation in young girls. Those at high risk of starting to smoke must be made aware of the addictive nature of tobacco and the myths that smoking can regulate mood disorders or help to control weight, or that quitting has negative health consequences. Additionally, although most of the informants were aware of the health consequences of smoking, a few suffered from the misapprehension that the adverse effects of smoking on health would take a long time to appear. It is essential therefore for healthcare professionals to raise public awareness of both the immediate and the chronic long-lasting health hazards of tobacco use.

With the rapid changes in social and economic structures in Hong Kong over recent years, more women are joining the workforce. However, at the same time these women might encounter more difficulty, negative emotions and additional stress in balancing their busy family and working lives [28]. Consistent with the Hong Kong Government’s Thematic Household Survey Report [19], our results revealed that negative emotions and stress were important factors in both smoking initiation and continued tobacco use among female smokers. For women with such problems, it is vital that healthcare professionals should focus on helping them understand the negative health consequences of smoking, and at the same time counselling them about alternative strategies for coping with negative emotions and stress. Most importantly, healthcare professionals should be offered relevant training to enhance their self-efficacy and confidence in promoting smoking cessation to female smokers.

### Implications for public health actions

Our findings highlight the urgent need to take public health action to prevent young girls and women from starting to smoke. A positive image of a healthy non-smoking female should be portrayed through education and publicity to the younger generation.

The findings indicated that many participants did not realise that there were female-specific health consequences of smoking. Campaigns are needed to raise public awareness of these negative consequences, such as higher rates of infertility, ectopic pregnancy, premature labour, infants with low birth-weight, sudden infant death syndrome, menstrual pain, early menopause, osteoporosis, cervical cancer and negative effects on the skin and appearance. Campaigns are also needed to increase the public’s support for women who reject smoking, and who avoid smokers and second-hand smoking exposure.

It is also important to break the use of tobacco as a tool for social networking. Tailor-made campaigns promoting smoking cessation and publicity targeting women are needed to help current smokers to quit. We must cultivate a social norm that does not tolerate smoking behaviour, that encourages non-smoking family members, friends and the general population to advise people to stop smoking in public areas and creates social pressure on and raises support for smokers to give up. Additionally, more promotion of existing smoking cessation services and more resources deployed to help females stop smoking, such as by setting up a women’s ‘quit line’, are essential.

Despite the comprehensive ban on tobacco advertising in Hong Kong, the tobacco industry still exploits loopholes to promote their products, such as different packaging of tobacco products and large, prominent and visually appealing cigarette displays at points of sale. It is therefore crucial for the government to mandate plain packaging of tobacco products to prohibit manufacturers from promoting sales through fancy and misleading designs on cigarette packets and displays. Moreover, there is an imperative need to adopt a new set of pictorial health warnings, especially for women and with stronger effects, and to change the warnings regularly to optimise their effectiveness and raise consumers’ awareness of smoking hazards and cessation. Additionally, to promote awareness of existing smoking cessation services, the quit line number should be highlighted on the plain packaging of tobacco products.

To tackle tobacco marketing campaigns, smoking prevention mass media campaigns and prevention policies prohibiting the advertising of tobacco products should be implemented. In particular, films and television projects with tobacco imagery or reference should become ineligible to apply for public subsidy. This can urge the private sector to comply with public health policies [29].

To enforce anti-smoking legislation, action should be taken to limit access to cigarettes, by restricting tobacco promotion and display at the points of sale, for example. Further, increasing tobacco tax massively is a tobacco control measure that has been proved to be most effective [20]. For the sake of public health, it is crucial to solicit more public support for legislation to increase tobacco tax substantially and regularly. Most importantly, non-smokers, healthcare professionals and anti-tobacco workers should work with the government to strengthen smoke-free legislation and policies, and to encourage the establishment of smoking cessation services to further reduce smoking prevalence to single figures, which would be the first step towards the ‘endgame’ for a smoke-free Hong Kong [30].

### Implications for future research

Despite the advantages of using focus group interviews, in-depth individual interviews could be conducted in future research to explore the more subjective experiences of individual participants. In-depth interviews can also provide complex textual descriptions of informants’ behaviour, attitudes and experiences and of the trajectories concerned with smoking. In addition, a future survey with a large sample of participants could help explore how socio-economic and demographic characteristics influence their behaviour, attitudes and experience related to smoking. Our findings have shown that there is a need to explore the issues of female smoking among Chinese population, so that effective gender-specific and culturally relevant smoking prevention and cessation programmes can be developed both to prevent further uptake of smoking and to motivate current female smokers to give up.

## Conclusions

This study has addressed a gap in the literature by examining the behaviour, attitudes and experiences of Chinese women related to smoking, not smoking and smoking cessation, an area of research that has been under-represented in the literature. The findings can be used in the development of smoking cessation interventions for Chinese female smokers. Most importantly, education, public policy, legislation and research all need to be geared towards preventing young girls and women from starting to smoke and motivating current smokers to quit. All such initiatives would serve as useful best-practice examples for China and other middle- and low-income regions.
